# The Reproductive Toxicity Valuation of Deoxynivalenol: An Integrated Study from Network Toxicology, Molecular Docking, Molecular Dynamics Simulation and Single-Cell RNA Sequencing

**DOI:** 10.3390/ijms27073068

**Published:** 2026-03-27

**Authors:** Liguo Dou, Yurou Tang, Siqi Yuan, Fan Xu, Yuanqing Wang, Qingjiao He, Jianye Yan

**Affiliations:** 1 College of Life Science and Technology, Central South University of Forestry and Technology, Changsha 410004, China; 20220707@csuft.edu.cn (L.D.); 20241200289@csuft.edu.cn (Y.T.); 20231200193@csuft.edu.cn (S.Y.); 20220552@csuft.edu.cn (F.X.); 2 School of Pharmacy, Hunan University of Medicine, Huaihua 418000, China; 3 Academy of Chinese Medical Sciences, Hunan University of Chinese Medicine, Changsha 410208, China; yanjy@hnucm.edu.cn; 4 Hunan Engineering Technology Research Center for Bioactive Substance Discovery of Chinese Medicine, Hunan University of Chinese Medicine, Changsha 410208, China

**Keywords:** deoxynivalenol, reproductive toxicity, network toxicology, molecular docking, molecular dynamics simulation, single-cell RNA sequencing

## Abstract

Deoxynivalenol (DON), a *Fusarium*-derived mycotoxin widely found in grain-based feed, has become a major global environmental contaminant. Reproductive toxicity is one of its most important toxic effects, yet systematic investigations covering both male and female reproductive injury remain limited. This study aimed to establish a combined strategy of network toxicology, molecular docking, molecular dynamics simulation, and single-cell RNA sequencing to evaluate the reproductive toxicity of DON. AKT1, EGFR, PIK3CA, PIK3R1, and SRC were identified as key targets involved in DON-induced reproductive injury. For testicular injury, the prolactin, Ras, HIF-1, and AGE-RAGE signaling pathways were closely associated with DON toxicity. For ovarian injury, the PI3K-Akt, HIF-1, prolactin, insulin, and AGE-RAGE signaling pathways were strongly implicated. Molecular docking demonstrated favorable binding affinities between DON and the hub targets, while molecular dynamics simulation further confirmed the stability of the DON–PIK3CA complex. Single-cell RNA sequencing analysis revealed that these five hub genes were highly expressed in both testicular (SRA667709:SRS3065430) and ovarian (SRA638923:SRS2797100) tissues. These findings deepen current understanding of DON-induced reproductive toxicity, provide new insights into the effects of environmental toxins on reproductive health, and offer a theoretical basis for future studies integrating DON exposure with in vivo validation of core targets and signaling pathways.

## 1. Introduction

Deoxynivalenol (DON), chemically designated as 3α,7α,15-trihydroxy-12,13-epoxytrichothec-9-en-8-one, is a type B trichothecene mycotoxin. It is a toxic secondary metabolite mainly produced by *Fusarium graminearum* and related fungal species and commonly contaminates cereals and animal feed, particularly wheat, barley, and maize [1]. Among trichothecene mycotoxins, DON is the most frequently detected in cereal grains and often occurs at relatively high concentrations. Owing to its strong resistance to heat, pressure, and acidic conditions, DON can readily persist in cereal-based foods and feedstuffs during processing, posing a substantial challenge to food safety [2]. In recent years, DON contamination has become a major global food safety concern. Concentrations ranging from 3800 to 6500 μg/kg have been reported in contaminated grains in North America [3]. DON has also been identified as the predominant mycotoxin in urine samples from swine production workers, as well as in litter and air samples collected from swine farms [4]. Biomonitoring studies further suggested that approximately 80% of the population had experienced DON exposure, as measured by urinary DON levels, used as a biomarker for exposure assessment [5]. These findings indicate that ingestion or inhalation of DON-contaminated food, feed, or environmental particles may pose a significant threat to human health. After exposure to DON-contaminated food, both humans and animals may develop a broad spectrum of toxic responses, including anorexia, vomiting, weight loss, neuroendocrine disorders, liver injury, renal damage, and immunosuppression [6]. Among these adverse effects, reproductive toxicity has attracted increasing attention. In males, DON exerts reproductive toxicity through multiple mechanisms. Experimental studies have shown that DON exposure severely impairs the integrity of the blood–testis barrier (BTB), thereby facilitating toxin infiltration into testicular tissue and inducing histopathological damage in the testes and epididymis, ultimately reducing sperm count and quality [7]. DON can also cause structural injury to the testes and epididymis, promote sperm morphological abnormalities, trigger testicular cell apoptosis, and impair sperm maturation and fertilization capacity [8]. In addition, DON disrupts the hypothalamic–pituitary–gonadal (HPG) axis, interferes with steroidogenesis and testosterone biosynthesis, and consequently contributes to reproductive dysfunction and male infertility [9]. The female reproductive system is also highly vulnerable to DON exposure. DON inhibits the proliferation of endometrial cells and ovarian granulosa cells and alters the synthesis of progesterone, testosterone, and estradiol in granulosa cells. These changes adversely affect oocyte maturation and embryonic development [10]. Previous studies have further demonstrated that DON exposure induces oocyte maturation defects, mitochondrial dysfunction, lysosomal damage, autophagy, endoplasmic reticulum stress, and dysfunction of the Golgi apparatus in porcine oocytes [11].

Although numerous studies have reported the reproductive toxicity of DON, systematic investigations into its overall reproductive toxic effects in both males and females remain limited. In particular, the core targets and signaling pathways underlying DON-induced reproductive toxicity have not been clearly defined, and the distribution of these targets across testicular and ovarian cell types remains poorly characterized. Network toxicology, as an extension of network pharmacology, is a systematic target-discovery approach that integrates multi-source databases to construct interaction networks among toxicant targets, disease targets, and biological processes, thereby uncovering toxicant–target–disease associations and potential toxicological mechanisms [12]. Molecular docking can further validate targets identified by network toxicology by simulating interactions between small molecules and target proteins and predicting binding affinity. This approach not only clarifies interactions between toxic compounds and disease-related targets but also facilitates the identification of key binding sites associated with pathogenic processes. Network toxicology and molecular docking shift toxicological research from the observation of macroscopic phenomena to the exploration of molecular interactions and system-level networks, thereby markedly improving the efficiency and depth of mechanistic studies. In addition, single-cell RNA sequencing (scRNA-seq) enables the characterization of core target expression patterns across distinct testicular and ovarian cell populations.

This study aimed to establish a combined strategy of network toxicology, molecular docking, molecular dynamics (MD) simulation, and scRNA-seq to explore the potential mechanisms underlying DON-induced reproductive toxicity. Core genes associated with DON-related reproductive injury were first screened and identified. Gene Ontology (GO) annotation and Kyoto Encyclopedia of Genes and Genomes (KEGG) pathway enrichment analyses were then performed to systematically reveal the key biological processes (BP), cellular components (CC), molecular functions (MF), and related signaling pathways involved. Molecular docking and MD simulation were further used to validate the binding characteristics of DON to the core targets. In addition, scRNA-seq was applied to characterize the expression and distribution patterns of these core targets in testicular and ovarian cell types. This research can clarify the underlying toxicity targets and related pathways of DON-induced reproductive injury by the combined strategy, provide a theoretical basis for the prevention and treatment of DON-related reproductive toxicity, and offer valuable support for the toxicological assessment and regulatory supervision of foodborne toxins.

## 2. Results

### 2.1. Potential Targets Analysis of DON

**Figure 1:**
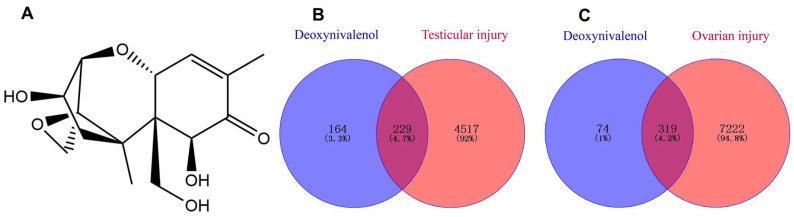
The structure of DON and Venn Diagrams of targets of DON and reproductive injury. (**A**) 2D structure of DON, (**B**) Venn Diagram of targets of DON and testicular injury, (**C**) Venn Diagram of targets of DON and ovarian injury.

The structure of DON and the targets of DON and reproductive injury were shown in Figure 1. The SMILES string of DON was imported into the Super-PRED database, yielding 112 potential targets. The SDF structure was submitted to the PharmMapper database, which predicted 299 potential targets. After merging the results from the two databases and removing duplicates in Excel, 393 potential DON-related targets were identified.

### 2.2. Potential Targets Analysis of Testicular Injury and Ovarian Injury

A total of 4618 targets associated with testicular injury and 7370 targets associated with ovarian injury were retrieved from the GeneCards database. In addition, 142 testicular injury-related targets and 217 ovarian injury-related targets were obtained from the OMIM database. After integrating the targets from both databases and removing duplicates, 4746 testicular injury-related targets and 7543 ovarian injury-related targets were ultimately identified.

### 2.3. Targets Analysis of DON-Induced Testicular and Ovarian Injury

The common targets involved in DON-induced testicular and ovarian injury are shown in Figure 1. As illustrated in Figure 1B, 229 overlapping targets were identified by intersecting DON targets with testicular injury-related targets using Venny 2.1.0. Similarly, as shown in Figure 1C, 319 common targets were obtained between DON targets and ovarian injury-related targets.

### 2.4. Establishment of PPI Network and Screening of Hub Genes

#### 2.4.1. Establishment of PPI Network

**Figure 2:**
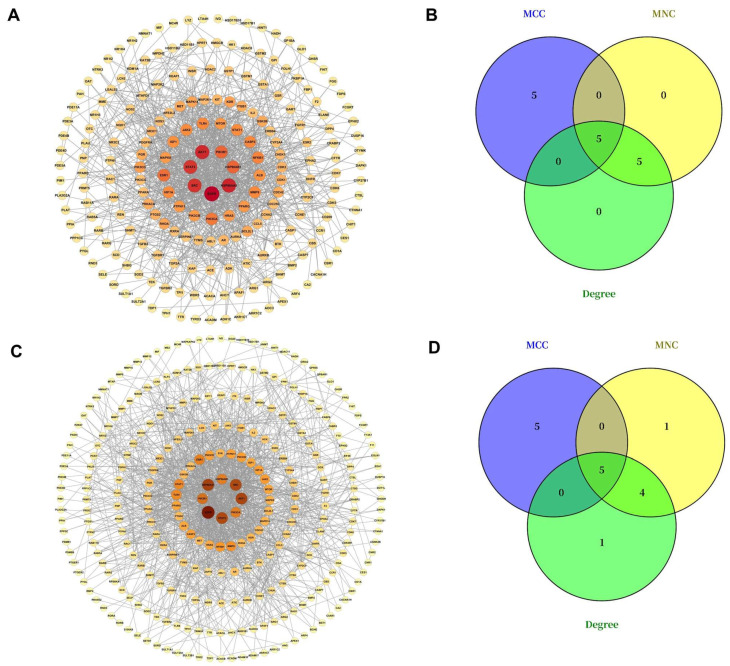
Network diagrams of targets related to DON-induced testicular injury and DON-induced ovarian injury. (**A**) PPI network diagram of common targets related to DON-induced testicular injury, (**B**) Venn Diagram of MCC, MNC and Degree of top 10 targets related to DON-induced testicular injury, (**C**) PPI network diagram of common targets related to DON-induced ovarian injury, (**D**) Venn Diagram of MCC, MNC and Degree of top 10 targets related to DON-induced ovarian injury.

The common targets were imported into the STRING database to construct the protein–protein interaction (PPI) networks for DON-induced testicular injury and DON-induced ovarian injury. The resulting data were further analyzed in Cytoscape 3.7.2 using the “Network Analyzer” function, with node size and color intensity adjusted according to degree values. Larger and darker nodes indicated greater importance within the network. The PPI networks are shown in Figure 2, where darker node colors indicate higher degrees. As shown in Figure 2A,C, EGFR, HSP90AA1, AKT1, SRC, and STAT3 exhibited relatively high degree values in the PPI networks of DON-induced testicular and ovarian injury.

#### 2.4.2. Screening of Hub Genes

Using the CytoHubba plugin, the top 10 candidate genes were ranked by Maximal Clique Centrality (MCC), Maximum Neighborhood Component (MNC), and Degree algorithms, and the detailed results are presented in Table 1. For DON-induced testicular injury, the top 10 genes identified by the MCC algorithm were JAK2, EGFR, PIK3CD, AKT1, SRC, PTPN11, PIK3CB, PIK3R1, HRAS, and PIK3CA; those identified by the MNC algorithm were STAT3, HSP90AA1, ESR1, PIK3CA, PIK3R1, AKT1, NFKB1, HSP90AB1, EGFR, and SRC; and those ranked by the Degree algorithm were SRC, ESR1, AKT1, HSP90AA1, PIK3R1, NFKB1, PIK3CA, HSP90AB1, STAT5, and EGFR. Intersection of the results from the three algorithms identified five hub genes: AKT1, EGFR, PIK3CA, PIK3R1, and SRC (Table 1; Figure 2A,B). Using the same approach, the top 10 genes associated with DON-induced ovarian injury identified by the MCC algorithm were PIK3CG, EGFR, PIK3R1, PTPN11, SRC, PIK3CB, PIK3CD, AKT1, JAK2, and PIK3CA; those identified by the MNC algorithm were EGFR, ESR1, HSP90AA1, AKT1, PIK3CA, PIK3R1, HSP90AB1, STAT3, TLR4, and SRC; and those ranked by the Degree algorithm were PIK3R1, STAT1, HSP90AA1, AKT1, STAT3, HSP90AB1, SRC, ESR1, EGFR, and PIK3CA. After intersecting the results from the three algorithms, the same five hub genes, namely AKT1, EGFR, PIK3CA, PIK3R1, and SRC, were identified (Figure 2C,D). These results indicated that the hub genes involved in DON-induced testicular injury and ovarian injury were identical.

### 2.5. GO Functional Annotation and KEGG Pathway Enrichment Analysis

#### 2.5.1. GO and KEGG Enrichment Analysis of DON-Induced Testicular Injury

**Figure 3:**
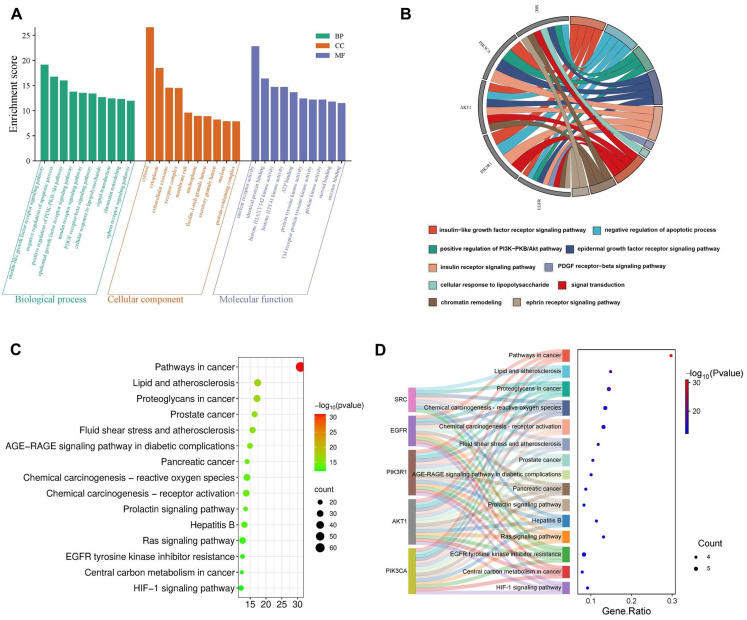
GO and KEGG enrichment analysis of DON-induced testicular injury. (**A**) The top 10 BPs, CCs and MFs in GO enrichment analysis, (**B**) The relationship between the core targets and biological process, (**C**) The top 15 KEGG pathways, (**D**) The relationship between the core targets and the top 15 KEGG pathways.

The DAVID database was used to analyze GO functional annotations and KEGG pathway enrichment analysis. The GO and KEGG results for targets associated with DON-induced testicular injury are shown in Figure 3. A total of 690 BPs, 77 CCs, and 229 MFs of DON-induced testicular injury were obtained. As shown in Figure 3A, the top 10 of BPs ranked by *p* values (*p* < 0.05) included negative regulation of apoptotic process, insulin-like growth factor receptor signaling pathway, positive regulation of the PI3K-Akt signaling pathway, insulin receptor signaling pathway, epidermal growth factor receptor signaling pathway, platelet-derived growth factor receptor-beta signaling pathway, cellular response to lipopolysaccharide, signal transduction, chromatin remodeling, and ephrin receptor signaling pathway. The top 10 enriched CCs were cytosol, cytoplasm, extracellular exosome, receptor complex, membrane raft, nucleoplasm, ficolin-1-rich granule lumen, secretory granule lumen, nucleus, and protein-containing complex. The top 10 enriched MFs included nuclear receptor activity, identical protein binding, histone H2AXY142 kinase activity, histone H3Y41 kinase activity, protein tyrosine kinase activity, ATP binding, transmembrane receptor protein tyrosine kinase activity, protein kinase activity, steroid binding, and enzyme binding. As shown in Figure 3B, each hub gene was involved in multiple BPs associated with DON-induced testicular injury. For instance, *AKT1* participated in the insulin-like growth factor receptor signaling pathway, epidermal growth factor receptor signaling pathway, negative regulation of the apoptotic process, insulin receptor signaling pathway, signal transduction, and chromatin remodeling.

A total of 172 KEGG pathways were enriched for DON-induced testicular injury. As illustrated in Figure 3C, the top 15 pathways included pathways in cancer, proteoglycans in cancer, lipid and atherosclerosis, prostate cancer, AGE-RAGE signaling pathway in diabetic complications, fluid shear stress and atherosclerosis, pancreatic cancer, chemical carcinogenesis–receptor activation, prolactin signaling pathway, hepatitis B, chemical carcinogenesis–reactive oxygen species, Ras signaling pathway, EGFR tyrosine kinase inhibitor resistance, central carbon metabolism in cancer, and HIF-1 signaling pathway. Among these, cancer pathways showed the highest degree of enrichment, the greatest statistical significance, and the largest number of enriched genes. Based on biological relevance, the major pathways associated with DON-induced testicular injury were the AGE-RAGE signaling pathway in diabetic complications, the prolactin signaling pathway, the Ras signaling pathway, and the HIF-1 signaling pathway. Figure 3D further demonstrated that the hub genes were involved in multiple enriched pathways, with AKT1 participating in all top 15 pathways.

#### 2.5.2. GO and KEGG Enrichment Analysis of DON-Induced Ovarian Injury

**Figure 4:**
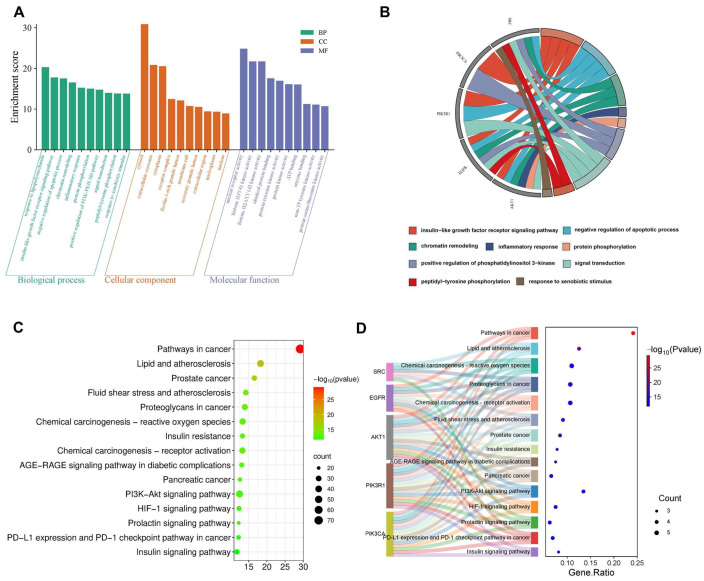
GO and KEGG enrichment analysis of DON-induced ovarian injury. (**A**) The top 10 BPs, CCs and MFs in GO enrichment analysis, (**B**) The relationship between the core targets and biological process, (**C**) The top 15 KEGG pathways, (**D**) The relationship between the core targets and the top 15 KEGG pathways.

The GO and KEGG enrichment results for targets associated with DON-induced ovarian injury are shown in Figure 4. As shown in Figure 4A, a total of 749 BPs, 93 CCs, and 244 MFs were identified. The top 10 enriched BPs ranked by *p* values (*p* < 0.05) included response to lipopolysaccharide, negative regulation of apoptotic process, insulin-like growth factor receptor signaling pathway, chromatin remodeling, inflammatory response, protein phosphorylation, positive regulation of the PI3K-Akt signaling pathway, signal transduction, peptidyl-tyrosine phosphorylation, and response to xenobiotic stimulus. The top 10 enriched CCs were cytosol, extracellular exosome, cytoplasm, receptor complex, membrane raft, ficolin-1-rich granule lumen, extracellular region, secretory granule lumen, nucleoplasm, and nucleus. The top 10 enriched MFs included nuclear receptor activity, histone H3Y41 kinase activity, histone H2AXY142 kinase activity, identical protein binding, protein tyrosine kinase activity, protein kinase activity, enzyme binding, non-receptor tyrosine kinase activity, ATP binding, and protein serine/threonine kinase activity. As shown in Figure 4B, all hub genes were involved in multiple biological processes related to DON-induced ovarian injury. For example, *AKT1* participated in the negative regulation of the apoptotic process, the insulin-like growth factor receptor signaling pathway, chromatin remodeling, the inflammatory response, protein phosphorylation, and signal transduction.

KEGG analysis identified 177 enriched pathways associated with DON-induced ovarian injury. As shown in 4C, the top 15 enriched pathways included pathways in cancer, prostate cancer, lipid and atherosclerosis, fluid shear stress and atherosclerosis, proteoglycans in cancer, chemical carcinogenesis–reactive oxygen species, AGE-RAGE signaling pathway in diabetic complications, insulin resistance, chemical carcinogenesis–receptor activation, pancreatic cancer, PI3K-Akt signaling pathway, PD-L1 expression and PD-1 checkpoint pathway in cancer, HIF-1 signaling pathway, prolactin signaling pathway, and insulin signaling pathway. Among these, pathways in cancer showed the highest enrichment level, greatest statistical significance, and largest number of enriched genes. Based on biological relevance, the major pathways associated with DON-induced ovarian injury were the PI3K-Akt signaling pathway, the AGE-RAGE signaling pathway in diabetic complications, the prolactin signaling pathway, the HIF-1 signaling pathway, and the insulin signaling pathway. As illustrated in Figure 4D, the hub genes were involved in multiple enriched pathways, with AKT1 participating in all top 15 pathways, similar to the findings for DON-induced testicular injury.

#### 2.5.3. AGE-RAGE Signaling Pathway in Diabetic Complications in DON-Induced Testicular Injury and Ovarian Injury

**Figure 5:**
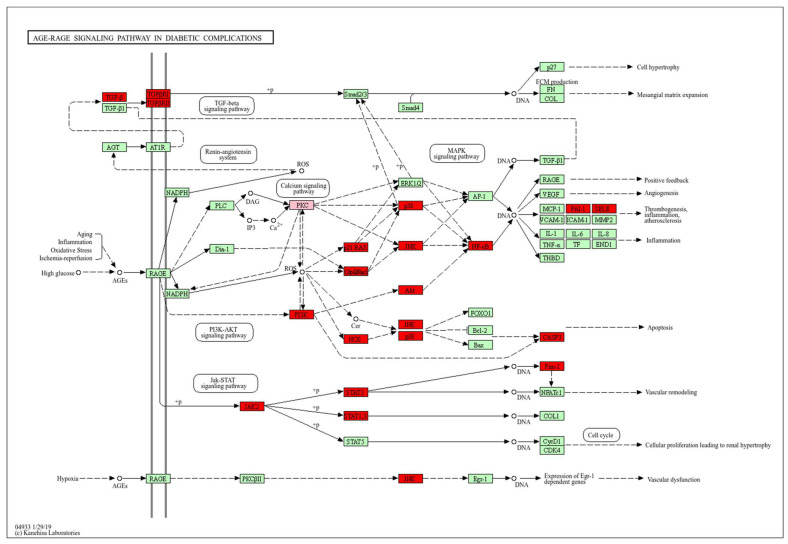
Potential targets and mechanism of DON-induced reproductive injury in AGE-RAGE signaling pathway in diabetic complications.

In the present study, the AGE–RAGE signaling pathway in diabetic complications was identified as a common pathway involved in both DON-induced testicular injury and ovarian injury. The targets enriched in this pathway are shown in Figure 5. As shown in Figure 5, red indicates the shared targets present in both DON-induced testicular injury and ovarian injury, whereas pink indicates the targets specifically enriched in DON-induced ovarian injury.

### 2.6. “DON-Hub Genes-Pathways-Reproductive Injury” Network Analysis

**Figure 6:**
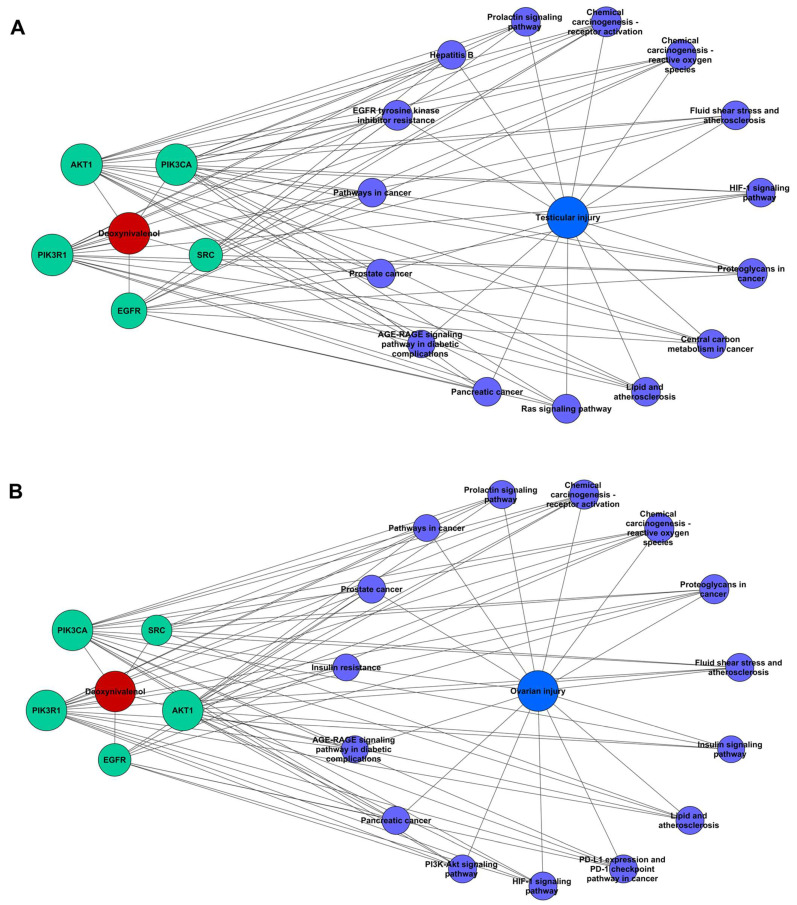
Relationship network among DON, key targets, top 15 KEGG pathways, and reproductive injury. (**A**) testicular injury, (**B**) ovarian injury.

Based on the KEGG enrichment results for DON-induced testicular injury and ovarian injury, the top 15 relevant pathways were selected to construct the “DON–hub genes–pathways–reproductive injury” network, thereby illustrating the relationships among DON, hub genes, pathways, and reproductive injury. The data were imported into Cytoscape 3.7.2 for visualization and topological analysis using the “Network Analyzer” function, in which node importance was evaluated based on degree. The resulting networks are shown in Figure 6, where red indicates DON, green indicates hub genes, purple indicates pathways, and blue indicates reproductive injury. As shown in Figure 6, the “DON–hub genes–pathways–testicular injury” network contained 22 nodes and 83 edges, whereas the “DON–hub genes–pathways–ovarian injury” network contained 22 nodes and 79 edges. These networks revealed the interactions among hub genes, enriched pathways, and reproductive injury. Notably, individual targets were involved in multiple pathways, and multiple targets converged on the same pathway, indicating that DON induces testicular and ovarian injury through a multi-target, multi-pathway mode of action.

### 2.7. The Results of Molecular Docking

**Figure 7:**
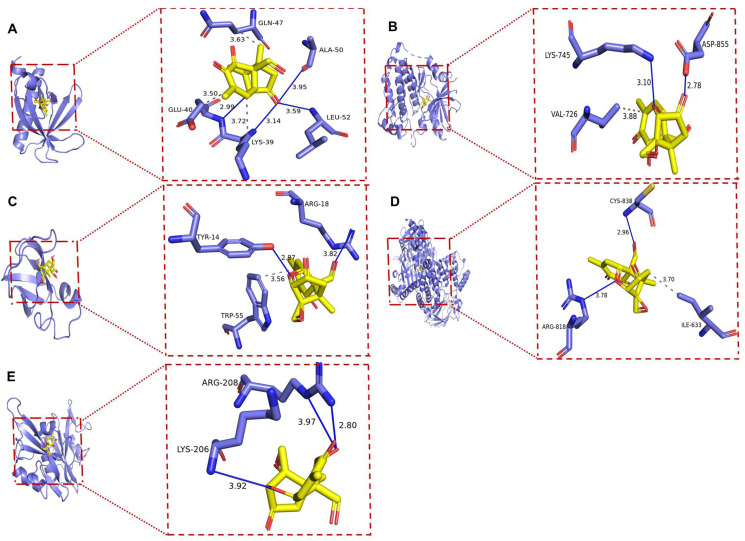
Molecular docking between DON and key targets. (**A**) DON-AKT1, (**B**) DON-EGFR, (**C**) DON-PIK3R1, (**D**) DON-PIK3CA, (**E**) DON-SRC. The gray dashed line represents hydrophobic interaction and the purple solid line represents hydrogen bond.

Molecular docking was performed between DON and the five hub targets (AKT1, EGFR, PIK3CA, PIK3R1, and SRC), using protein structures from the PDB database as receptors and DON as the ligand. Docking was conducted using AutoDock Vina 1.12, and the binding energies are summarized in Table 2 and Figure 7. As shown in Table 2, the binding energies of DON with AKT1, EGFR, PIK3CA, PIK3R1, and SRC were −6.1, −7.5, −7.6, −5.9, and −5.2 kcal/mol, respectively. Lower docking energy indicates stronger binding affinity. All docking energies were below −5.0 kcal/mol, suggesting favorable binding of DON to all five core targets. Among them, DON showed the strongest binding affinity toward PIK3CA. As shown in Figure 7A, DON formed hydrophobic interactions with LYS39 (3.72 Å), GLU40 (3.50 Å), and GLN47 (3.63 Å) in *AKT1*, as well as hydrogen bonds with LYS39 (3.14 Å), GLU40 (2.99 Å), ALA50 (3.95 Å), and LEU52 (3.59 Å). In EGFR (Figure 7B), DON formed a hydrophobic interaction with VAL726 (3.88 Å) and hydrogen bonds with LYS745 (3.10 Å) and ASP855 (2.78 Å). In PIK3CA (Figure 7C), DON formed a hydrophobic interaction with ILE633 (3.70 Å), together with hydrogen bonds with ARG818 (3.78 Å) and CYS838 (2.96 Å). In PIK3R1 (Figure 7D), DON formed a hydrophobic interaction with TRP55 (3.56 Å) and hydrogen bonds with TYR14 (2.87 Å) and ARG18 (3.82 Å). In SRC (Figure 7E), DON formed hydrogen bonds with LYS206 (3.92 Å) and ARG208 (2.80 and 3.97 Å). Overall, hydrogen bonds and hydrophobic interactions were the predominant binding modes between DON and the hub targets.

To validate the feasibility of the docking, the original co-crystallized ligands were extracted and redocked into the proteins. The RMSD between the redocked pose and the native conformation was calculated. The RSMD values of redocking of PIK3CA, AKT1, EGFR and SRC were 0.557 Å, 0.243 Å, 0.385 Å and 0.681 Å, respectively, which indicated that the docking method was reliable.

### 2.8. Molecular Dynamics Simulation

**Figure 8:**
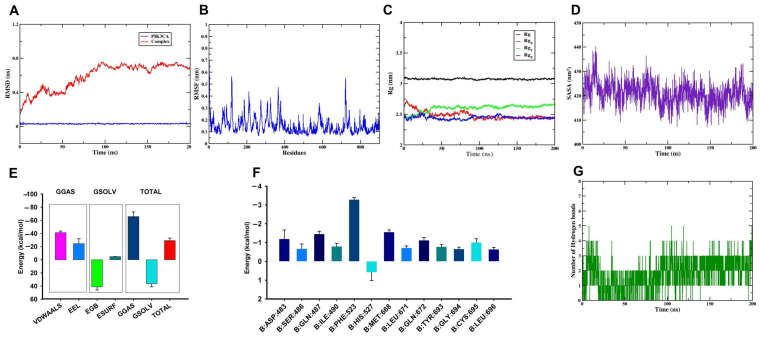
Molecular dynamics simulation between DON and PIK3CA protein. (**A**) RMSD, (**B**) RMSF, (**C**) Rg, (**D**) SASA, (**E**) Energy attribute breakdown diagram of DON-PIK3CA, (**F**) Energy amino acid decomposition diagram of DON-PIK3CA, (**G**) Number of hydrogen bonds in DON-PIK3CA. VDWAALS, van der Waals energy; EEL, Electrostatic energy; EGB, polar solvation energy; ESURF, nonpolar solvation energy; GGAS, total gas phase free energy, GGAS, VDWAALS + EEL; GSOLV, total solvation free energy, EGB + ESURF; TOTAL, GSOLV + GGAS.

MD simulation of the DON–PIK3CA complex was performed based on the optimal binding affinity observed in the molecular docking analysis. The results are shown in Figure 8. Root-mean-square deviation (RMSD) is a key indicator used to assess the stability and structural convergence of protein–ligand complexes. As illustrated in Figure 8A, the RMSD value of the DON–PIK3CA complex stabilized at approximately 0.8 nm after 100 ns, indicating that the complex reached equilibrium and remained stable during the simulation. Root mean square fluctuation (RMSF) reflects the flexibility of individual amino acid residues. As illustrated in Figure 8B, the residue fluctuations of *PIK3CA* ranged from 0.1 to 0.6 nm, suggesting relatively good local stability throughout the simulation. The radius of gyration (Rg), which reflects structural compactness, showed only minor fluctuations in the RgX, RgY, and RgZ components, whereas the overall Rg value remained stable (Figure 8C). In addition, the solvent accessible surface area (SASA), an indicator of protein folding and structural stability, remained between 410 and 440 nm^2^ (Figure 8D), further supporting the stable conformation of the DON–PIK3CA complex.

The molecular mechanics/generalized Born surface area (MM/GBSA) method was further used to estimate the binding free energy of the DON–PIK3CA complex, and the calculations were performed using the Gmx_MMPBSA tool. As shown in Figure 8E, both electrostatic and van der Waals interaction energies were below 0, indicating that both contributed favorably to ligand binding. After simulation, the total binding free energy was −29.23 ± 3.71 kcal/mol, suggesting a stable interaction between DON and PIK3CA. The residue-wise energy decomposition results are shown in Figure 8F, where PHE-523, MET-668, GLN-487, ASP-483, GLN-672, and CYS-695 showed relatively large energy contributions, with PHE-523 exhibiting the greatest contribution. In addition, hydrogen-bond analysis revealed stable interactions between DON and PIK3CA throughout the 200 ns simulation. As shown in Figure 8G, the number of hydrogen bonds ranged from 0 to 5, with an average of 1.81. Collectively, these results indicate that DON can bind stably to PIK3CA.

### 2.9. Results of Single-Cell RNA Sequencing

**Figure 9:**
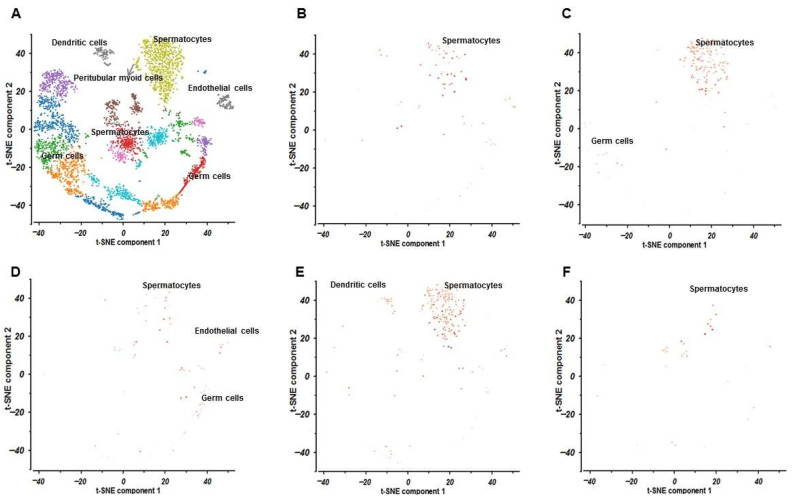
Results of single-cell RNA sequencing in testicle (SRA667709:SRS3065430). (**A**) t-SNE cluster analysis results. (**B**–**F**) Expression and analysis of core genes including AKT1, EGFR, PIK3CA, PIK3R1, and SRC in testicle (SRA667709:SRS3065430).

**Figure 10:**
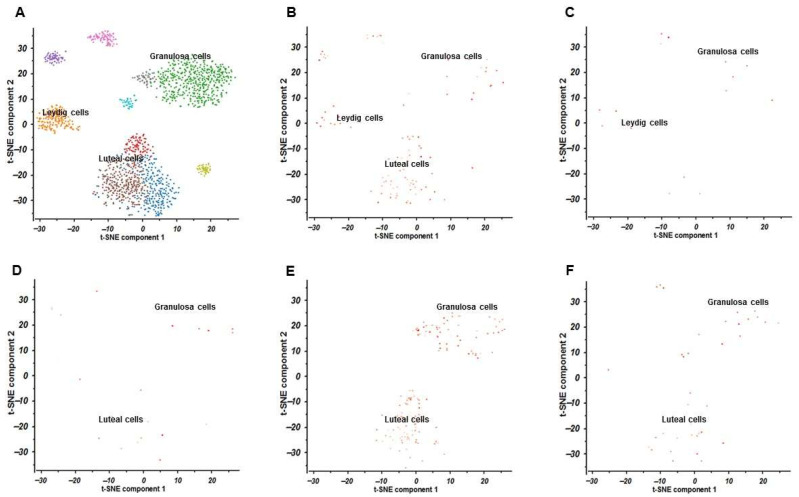
Results of single-cell RNA sequencing in ovary (SRA638923:SRS2797100). (**A**) t-SNE cluster analysis results. (**B**–**F**) Expression and analysis of core genes including AKT1, EGFR, PIK3CA, PIK3R1, and SRC in ovary (SRA638923:SRS2797100).

The scRNA-seq data for the testis (SRA667709:SRS3065430) and the ovary (SRA638923:SRS2797100) were obtained from the PanglaoDB database. The t-distributed stochastic neighbor embedding (t-SNE) clustering results for the testis and ovary are shown in Figure 9 and Figure 10, respectively. In this study, the testis dataset originated from *Homo sapiens*, whereas the ovary dataset was derived from *Mus musculus*. Based on scRNA-seq data, testicular cells (SRA667709:SRS3065430) were classified into five clusters: spermatocytes, germ cells, endothelial cells, dendritic cells, and peritubular myoid cells. Ovarian cells (SRA638923:SRS2797100) were divided into three clusters, including luteal cells, Leydig cells, and granulosa cells. The expression patterns of the five core genes, namely AKT1, EGFR, PIK3CA, PIK3R1, and SRC, are shown in Figure 9B–F for the testis dataset and Figure 10B–F for the ovary dataset. In testicular tissue, AKT1 was highly expressed in spermatocytes; EGFR was mainly expressed in spermatocytes and germ cells; PIK3CA was predominantly enriched in spermatocytes, endothelial cells, and germ cells; PIK3R1 showed relatively high expression in dendritic cells and spermatocytes; and SRC was mainly expressed in spermatocytes. In ovarian tissue, AKT1 showed relatively high expression in granulosa cells, Leydig cells, and luteal cells; EGFR was expressed at low levels in granulosa cells and Leydig cells; PIK3CA showed scattered expression in granulosa cells and luteal cells; PIK3R1 was mainly enriched in granulosa cells and luteal cells; and SRC was actively expressed in granulosa cells and luteal cells.

## 3. Discussion

Given the serious threat DON poses to food safety and human health, increasing attention has been directed toward its toxic effects. Conventional toxicological studies mainly rely on in vitro cell experiments and in vivo animal models. Although these approaches can reflect the actual toxic effects of DON, they are often insufficient to meet the growing need for rapid assessment of the increasing number of chemical toxins present in the food environment. In recent years, network toxicology and molecular docking have become increasingly mature and have been widely applied in the investigation of toxicological mechanisms. Network toxicology enables rapid identification of molecular interaction networks between diseases and emerging chemical toxins by integrating bioinformatics, chemistry, pharmacology, and computational science. In parallel, molecular docking and molecular dynamics simulation provide valuable tools for modeling biomolecular interactions and further validating the binding properties between toxins and candidate targets. Therefore, the combination of network toxicology, molecular docking, and molecular dynamics simulation not only offers an efficient and accurate strategy for elucidating mechanisms underlying toxin action but also provides a useful framework for evaluating the toxicological effects of other foodborne toxins that remain insufficiently studied.

### 3.1. Network Toxicology

In the present study, network toxicology identified 229 targets associated with DON-induced testicular injury and 319 targets associated with DON-induced ovarian injury. Notably, the hub genes associated with both types of reproductive injury were identical, namely AKT1, EGFR, PIK3CA, PIK3R1, and SRC, suggesting that these genes may serve as shared core regulators of DON-induced reproductive toxicity.

AKT1 has been reported to protect germ cells and inhibit germ cell apoptosis in several animal models of testicular injury, mainly by promoting spermatogonial stem cell survival and accelerating self-renewal. AKT1 deficiency in the testis has been associated with mitochondrial dysfunction and delayed Sertoli cell maturation, ultimately leading to premature germ cell apoptosis. In addition, Akt activation has been shown to alleviate cyclophosphamide-induced testicular injury and sperm DNA damage [13]. As a key regulator of cell survival, proliferation, and growth, AKT1 also plays an important role in ovarian pathology. In ovarian cancer (OC), the E17K mutation in the pleckstrin homology (PH) domain of AKT1 is a well-recognized hotspot mutation that activates the PI3K-Akt pathway, enhances cell migration and oncogenic potential, and cooperates with other oncogenic signals to promote malignant transformation [14].

EGFR, a transmembrane glycoprotein of the ErbB receptor tyrosine kinase family, is also closely associated with reproductive function. Members of the EGFR family regulate germ cell development, and previous studies have shown that EGFR signaling is essential for maintaining the balance between apoptosis and proliferation in the testis and for regulating spermatogenesis. Altered EGFR expression or activity has also been implicated in the invasiveness of testicular tumors [15].

EGFR expression has been reported to be significantly higher in OC than in normal ovarian tissue [16]. EGFR overexpression can promote OC cell proliferation through PI3K/AKT, MAPK, and STAT signaling pathways, and its amplification or overactivation is closely associated with enhanced proliferative and migratory capacities of OC cells [16].

PIK3CA, which encodes a catalytic subunit of phosphoinositide 3-kinase (PI3K), phosphorylates phosphatidylinositol and functions as a key component of the PI3K-Akt signaling pathway. Previous studies have suggested that Tripterygium glycosides may induce male reproductive toxicity by downregulating PIK3CA expression, indicating that PIK3CA may represent an important target in DON-induced testicular injury. In addition, PIK3CA mutation or amplification can activate the PI3K-Akt pathway in multiple human cancers and plays a critical role in the pathogenesis of OC. For example, ectopic expression of the tumor suppressor miR-337-3p has been shown to inhibit proliferation of epithelial ovarian cancer (EOC) cells, induce G0/G1 cell cycle arrest, and promote apoptosis [17]. Similarly, upregulation of miR-381 suppresses proliferation in certain EOC subtypes by reducing PIK3CA mRNA and p110α expression [18]. Aberrant activation of the PI3K-Akt pathway has also been widely reported in ovarian carcinoma, and Akt activation is particularly common in high-grade, late-stage serous OC [19].

PIK3R1 encodes p85α, the major regulatory subunit of class I PI3K, which negatively regulates the catalytic activity of p110α and directly interacts with PTEN to enhance its lipid phosphatase activity. Aberrant expression of PIK3R1 has been associated with increased proliferation, enhanced invasion, and reduced apoptosis in tumors. Mutations in PIK3R1 can weaken the inhibitory effect of p85α on p110α, thereby enhancing PI3K signaling. Therefore, PIK3R1 is generally considered to have tumor-suppressive potential, and its alterations have been linked to multiple cancer types. Although *PIK3R1* mutations are relatively uncommon in OC, they have been reported in endometrioid and clear cell subtypes. In addition, copy number loss and reduced PIK3R1 mRNA expression are characteristic of aggressive high-grade OC, suggesting that PIK3R1 downregulation may contribute to ovarian carcinogenesis [20]. Notably, autosomal dominant combined primary immunodeficiency caused by PIK3CD and PIK3R1 mutations has also been associated with lymphoproliferative disorders and malignancies, including testicular yolk sac tumors [21].

SRC, encoded by the *Src* gene, is a non-receptor tyrosine kinase belonging to the *Src* family kinases (SFKs). As a representative member of this family, c-*Src* has been widely implicated in tumor initiation and progression. Overexpression or aberrant activation of c-*Src* is frequently observed in multiple cancer types, and constitutive activation of c-*Src* by oncogenic signals can lead to pathological changes and enhanced tumor aggressiveness [22]. In OC, TRIM50 has been reported to function as a tumor suppressor by downregulating Src expression. It also markedly inhibited tumor growth in a xenograft model by suppressing Src kinase activity [23].

KEGG enrichment analysis further showed that the prolactin signaling pathway, Ras signaling pathway, HIF-1 signaling pathway, and AGE–RAGE signaling pathway in diabetic complications were closely associated with DON-induced testicular injury. In contrast, the PI3K-Akt signaling pathway, HIF-1 signaling pathway, prolactin signaling pathway, insulin signaling pathway, and AGE–RAGE signaling pathway in diabetic complications were strongly linked to DON-induced ovarian injury.

Prolactin (PRL) is a 23 kDa protein secreted mainly by lactotroph cells in the anterior pituitary and exerts its effects through binding to the prolactin receptor (PRLR). The JAK2/STAT5 pathway is considered the principal downstream signaling cascade of PRLR. Upon PRL binding, PRLR undergoes dimerization and conformational change, which promotes kinase recruitment to the intracellular domain and initiates downstream signaling [24]. Because the intracellular domain of PRLR lacks intrinsic kinase activity, it depends on cytoplasmic kinases to mediate signal transduction [25]. Previous studies have indicated that both hypo- and hyperprolactinemia may impair male fertility and reduce sperm quality by disturbing steroid metabolism and ribosome biogenesis, thereby decreasing testosterone production and promoting germ cell apoptosis in the testis [26]. PRL and PRLR have also been identified as important regulators in OC progression. Increased PRLR expression has been associated with elevated OC risk, suggesting a tumor-promoting role of PRL. Mechanistically, PRL can activate the JAK/STAT pathway, thereby promoting tumor cell survival and growth through transcriptional regulation and anti-apoptotic effects [27].

Ras proteins are members of the small GTPase family and play central roles in regulating cell proliferation, migration, apoptosis, and survival. Mutant RAS is among the most common oncogenic alterations in human cancers, occurring in approximately 19% of tumors. Upon activation, RAS exchanges GDP for GTP via guanine nucleotide exchange factors (GEFs), and the active RAS-GTP complex subsequently interacts with downstream effectors to regulate diverse cellular processes. The two major downstream signaling axes are the RAS–RAF–MEK–ERK and RAS–PI3K–AKT–mTOR pathways [28]. Among them, the RAS–RAF–MEK–ERK cascade, a key branch of the MAPK pathway, is essential for tumor cell survival and development. Constitutive activation of RAS can lead to dysregulation of MAPK signaling, which is closely associated with multiple human diseases, especially cancer. Previous studies have shown that the RAS–RAF–MEK–ERK pathway is involved in the formation of testicular germ cell tumors, suggesting that DON may contribute to testicular injury through Ras signaling [29]. In addition, chlorpyrifos exposure in male rats was reported to cause loss, disorganization, and sloughing of spermatogenic cells, as well as cytoplasmic vacuolization and cytoskeletal disruption in Sertoli cells, partly through dysregulation of the Ras pathway [30]. Activation of Ras signaling has been reported to prevent cellular senescence and improve ovarian reserve in aged females [31].

HIF-1 (hypoxia-inducible factor 1) is a transcription factor activated under hypoxic conditions and serves as a key regulator of cellular adaptation to low-oxygen microenvironments. It mediates a range of biological responses, including angiogenesis, migration, proliferation, and metabolic reprogramming, by inducing the expression of multiple downstream genes. Owing to its central role in hypoxia adaptation, HIF-1 is highly expressed in many tumor types. Previous studies have shown that asthma-induced hypoxia can activate the HIF-1 pathway and increase the expression of IL-6, STAT3, and HIF-1α, thereby promoting inflammation, impairing testicular function and sperm quality, and ultimately contributing to male infertility [32]. In addition, isoalantolactone has been reported to exert anti-testicular cancer effects through mechanisms associated with ferroptosis and HIF-1 signaling [33]. In OC, cancer-associated fibroblasts (CAFs) can activate HIF-1α in chemotherapy-resistant ovarian clear cell carcinoma subpopulations, suggesting that diffusible stromal signals, rather than hypoxia alone, may contribute to HIF-1α activation in this setting [34]. Advanced glycation end products (AGEs) are generated through non-enzymatic reactions between glucose and amino groups in proteins, lipids, and nucleic acids under hyperglycemic conditions. By binding to their receptor, RAGE, AGEs regulate a variety of cellular responses and activate multiple profibrotic pathways. High glucose can stimulate reactive oxygen species production, neurohumoral responses, growth factor cascades such as TGF-β/Smad3 and PDGF, and the release of pro-inflammatory cytokines, chemokines, and AGEs. Activation of the AGE–RAGE axis further promotes several downstream pathways, including ERK signaling, fibrotic growth factor signaling, and NF-κB-dependent collagen synthesis [35]. In males with diabetes, excessive AGE accumulation occurs in the testes, where both AGEs and RAGE are widely distributed. AGEs not only induce oxidative stress through protein cross-linking, but also activate downstream pathways such as CDC42 and AKT1, leading to mitochondrial superoxide overproduction, inhibition of glyceraldehyde-3-phosphate dehydrogenase, and oxidative damage to testicular tissue and sperm, ultimately contributing to male infertility [36]. In vitro studies have further shown that AGEs suppress testosterone production in Leydig cells by increasing GRP78 and CHOP expression [37]. In females, elevated AGE levels and increased RAGE expression have been reported in polycystic ovary syndrome (PCOS). AGEs can impair steroidogenesis and follicular development, promote hyperandrogenism, and increase reactive oxygen species generation, thereby aggravating oxidative stress and disrupting cellular metabolism [38,39].

The PI3K-Akt pathway is another major signaling cascade implicated in ovarian injury. As a central downstream effector of Akt, mTOR integrates multiple upstream signals to promote cell growth, survival, and metabolic regulation through the PI3K/AKT/mTOR axis. This pathway is among the most frequently altered signaling pathways in human cancers. In OC, dysregulation of PI3K/AKT/mTOR signaling has been associated with genomic alterations in PTEN, AKT, PIK3CA, PIK3R1, mTOR, TSC1, and TSC2. In particular, amplification or mutation of *PIK3CA* can lead to aberrant activation of this pathway [40]. It has been reported that more than 20% of ovarian cancer cases harbor genomic alterations in PIK3CA, supporting its role as an oncogenic driver in OC [41]. Beyond tumor progression, the PI3K-Akt pathway is also closely involved in cell survival and inflammatory regulation, and reduced activity of this pathway may promote apoptosis and inflammatory responses. In a mouse model of PCOS, the PI3K/AKT pathway was found to be suppressed [42].

Insulin is a peptide hormone secreted by pancreatic β-cells that binds to cell surface receptors and triggers receptor tyrosine autophosphorylation, thereby activating intracellular signaling cascades. Insulin signaling mainly branches into two major pathways: the metabolic PI3K-Akt pathway and the mitogenic MAPK-ERK pathway. Insulin resistance is considered a central feature of PCOS pathogenesis. Insulin receptors are abundantly expressed in ovarian stromal and follicular cells, where insulin directly regulates steroidogenesis and ovulation [43]. In PCOS, decreased tyrosine phosphorylation of the insulin receptor and insulin receptor substrate, together with increased serine phosphorylation, can impair insulin signaling transduction and contribute to post-receptor defects and insulin dysfunction [44]. Previous studies have also shown that PCOS granulosa cells exhibit increased mTOR levels and reduced IRS-1 mRNA expression, while berberine may alleviate PCOS by regulating the IRS-1/mTOR signaling pathway [45].

Notably, several targets and pathways identified by network toxicology, including those discussed above [27,28,29,40], are closely associated with cancer-related processes. As reproductive cancers are also important forms of reproductive system injury, it is reasonable that “pathways in cancer” showed a high level of enrichment in the KEGG analysis.

Although the same hub genes and several common pathways were identified for both testicular and ovarian injury, our results also revealed important differences between the two. For example, NFKB1 and STAT1 were identified as differential genes among the top-ranked nodes in the PPI analysis. In addition, the Ras signaling pathway was one of the major pathways associated with DON-induced testicular injury, whereas the insulin signaling pathway was more prominently associated with DON-induced ovarian injury. These findings suggest that, despite shared molecular mechanisms, DON may exert distinct regulatory effects in male and female reproductive tissues.

The identified hub targets could be evaluated for biological relevance to reproductive tissues by GeneCards database. Through searching the hub genes (AKT1, EGFR, PIK3CA, PIK3R1, and SRC) in the GeneCards database, all of them were observed to be highly expressed in reproductive tissues including ovary, uterus, prostate and testis. The disorders of these genes will lead to reproductive damaging diseases including ovarian cancer, endometrial cancer, prostate cancer, testicular cancer, and so on. The published studies [14,15,18,20,23] also confirm the roles of the hub genes in reproductive tissue function, reproductive physiology and related diseases.

### 3.2. Molecular Docking and MD

Molecular docking and MD simulation were used to evaluate the binding characteristics of DON toward key targets associated with reproductive injury. Docking analysis showed that DON exhibited favorable binding affinities with all five hub targets, with binding energies below −5.0 kcal/mol. Among them, PIK3CA showed the strongest binding affinity and was therefore selected for subsequent MD simulation. Over the 200 ns simulation, the DON–PIK3CA complex maintained a stable conformation, as evidenced by RMSD, RMSF, Rg, SASA, and hydrogen bond analyses. Moreover, the calculated binding free energy (Gobind) further confirmed the strong interaction between DON and PIK3CA. The molecular dynamics simulation of DON-PIK3CA was a further validation of its molecular docking.

### 3.3. Single-Cell-RNA Sequencing Study

Single-cell-RNA Sequencing further showed that the five hub genes were expressed in both testicular (SRA667709:SRS3065430) and ovarian (SRA638923:SRS2797100) tissues. In the testis, these hub genes were mainly expressed in spermatocytes, germ cells, endothelial cells, and dendritic cells. In the ovary, they were predominantly expressed in granulosa cells, Leydig cells, and luteal cells. Although the Panglao DB datasets are mainly derived from human and mouse samples and remain limited by incomplete species coverage, the cell type-specific localization of hub genes identified in testicular and ovarian tissues still provides valuable insight into the potential cellular basis of DON-induced reproductive toxicity.

This study explored the potential targets and pathways involved in DON-induced reproductive toxicity by integrating network toxicology, molecular docking, molecular dynamics simulation, and single-cell-RNA-seq. The results suggested that DON may exert reproductive toxicity through a multi-target, multi-pathway mode of action. These findings not only deepen current understanding of DON toxicology but also provide a theoretical basis for future prevention and intervention strategies against DON-induced reproductive injury. However, because the major findings of this study were derived from computational prediction and bioinformatics analyses, further validation through in vitro and in vivo experiments is still required to confirm their biological reliability and practical relevance. Given the widespread occurrence of DON in food and the environment, strengthened monitoring and effective control of DON contamination are also essential for improving food and environmental safety.

## 4. Materials and Methods

### 4.1. Targets Collection for Deoxynivalenol

“Deoxynivalenol” was searched in the PubChem database (https://pubchem.ncbi.nlm.nih.gov/) to obtain its SMILES string and download its SDF structure. The PubChem CID of deoxynivalenol is 40024. The Super-PRED (https://prediction.charite.de/, collected in 9 February 2025) and Pharm Mapper (http://www.lilab-ecust.cn/pharmmapper/submitfile.html, collected in 10 February 2025) databases were used to identify potential targets for DON. All targets were then standardized using the Uniports database (https://www.uniprot.org/, accessed in 10 February 2025). After integrating the results from the two databases and removing duplicates, the final set of potential DON-related targets was obtained.

### 4.2. Targets Screening for Diseases

The OMIM (https://www.omim.org/, collected in 9 February 2025) and GeneCards (https://genecards.org/, collected in 8 February 2025) databases were used to identify genes associated with testicular and ovarian injuries using the keywords “Testicular injury” and “Ovarian injury”. In OMIM database, the keywords were searched in Gene Map and the Excel files were downloaded. The genes with “Approved Symbols” were retained. In GeneCards database, the genes with “Relevance scores” over 0.5 were selected. After integrating the targets retrieved from the two databases and removing duplicates, the final disease-related targets for testicular injury and ovarian injury were obtained.

### 4.3. Identification of Targets of DON-Induced “Testicular Injury” and “Ovarian Injury”

The common targets between DON-related targets and disease-related targets were identified using Venny 2.1.0 (https://bioinfogp.cnb.csic.es/tools/venny/, 12 February 2025). The overlapping targets between DON and testicular injury were defined as targets associated with DON-induced testicular injury, whereas the overlapping targets between DON and ovarian injury were defined as targets associated with DON-induced ovarian injury.

### 4.4. Construction of PPI Network and Acquisition of Hub Genes

The common targets between DON targets and disease-related targets for both “Testicular injury” and “Ovarian injury” were imported separately into the STRING 12.0 database (https://cn.string-db.org/, accessed in February 2025), with the species set to “Homo sapiens”. The full functional PPI networks were constructed with a confidence score of 0.7 and disconnected nodes hidden. The resulting network data were imported into Cystoscope 3.7.2 for visualization and further analysis. The Cohiba plugin was then used to identify the top 10 key genes based on the MCC, MNC, and Degree algorithms. Hub genes were defined as the intersecting genes identified by all three algorithms.

### 4.5. GO and KEGG Enrichment Analyses

The DAVID database (https://david.ncifcrf.gov/, accessed in 13 February 2025) was used for GO analyses and KEGG pathway enrichment analyses of targets associated with DON-induced testicular and ovarian injury. The enrichment analyses were visualized on the Bioinformatics platform (http://www.bioinformatics.com.cn/, accessed in 13 February 2025) using histograms, bubble plots, and chord diagrams.

### 4.6. Construction of “DON-Hub Genes-Pathways-Reproductive Injury” Network

Based on the identified hub genes and KEGG enrichment results, an Excel file was created to define the relationships among DON, hub genes, pathways, and reproductive injury. These data were then imported into Cystoscape 3.7.2 to construct the “DON–hub genes–pathways–testicular injury” and “DON–hub genes–pathways–ovarian injury” networks.

### 4.7. Molecular Docking

DON was used as the ligand for molecular docking with the proteins encoded by the hub genes. The 2D SDF structure of DON from PubChem (https://pubchem.ncbi.nlm.nih.gov/, accessed in 26 February 2025) was optimized in Chem3D 19.0 and saved in MOL2 format. The Uniports database (https://www.uniprot.org/, accessed in 26 February 2025) was adopted to obtain the Entry names of the hub genes. Next, these Entry names were searched in the PDB database (https://www.rcsb.org/) to select protein structures with Homo sapiens, X-ray diffraction data, ligands, high resolutions, and relatively complete structures. The PDB files were downloaded, and molecular docking of DON with the core targets was performed using Auto Dock Vina 1.12. The water molecules and ligands were removed from the initial protein structure before molecular docking. The ligands were added hydrogen atoms using Auto Dock Tools. A docking box of the appropriate size was set to ensure the protein was completely covered. A configuration file containing the docking box information was output to facilitate further docking operations. Docking was performed, and the binding affinity was calculated. The interactions were obtained by Protein-Ligand Interaction Profiler (PLIP, https://plip-tool.biotec.tu-dresden.de/plip-web/plip/, accessed in 19 March 2025) and visualized by PyMOL2.3.0. To validate the feasibility of the docking, the original co-crystallized ligands were extracted from the proteins by PyMOL2.3.0. The water molecules were removed from the protein and the original co-crystallized ligands were hydrogenated before redocking. The ligands were redocked to the proteins with the same method. The RMSD between the redocked pose and the native conformation was calculated by PyMOL 2.3.0.

### 4.8. MD Simulation

MD simulation of DON-PIK3CA was performed using GROMACS 2023. The Amber ff99SB-ildn force field was applied to the protein, and the General Amber Force Field (GAFF) was used for DON. The RESP charges of ligand (DON) were obtained through Multiwfn 3.8 and Gaussian09w software packages. The complex was placed in a cubic water box (10 × 10 × 10 nm^3^) with a minimum distance of 1.2 nm from the protein to the box edge and solvated using the TIP3P water model. A total of 28 chloride ions were added to neutralize the system. Electrostatic interactions were calculated using the particle mesh Ewald (PME) method. Energy minimization was performed using the steepest descent algorithm with a maximum of 50,000 steps. The cutoff distances for both Coulombic and van der Waals interactions were set to 1.0 nm. A 200 ns MD simulation was then carried out under NPT conditions at 300 K and 1 bar. Temperature and pressure were maintained using the V-rescale thermostat and Berendsen barosta, respectively. The binding free energy of the DON–PIK3CA complex was further calculated using the MM/GBSA method.

### 4.9. Single-Cell RNA-Seq

Using the “Samples” module in the Panglao DB database (https://panglaodb.se/, accessed in 10 August 2025), datasets were retrieved with “Mouse & Human” and “Tissue” set as filtering criteria. The datasets of Testicle (SRA667709:SRS3065430) and Ovary (SRA638923:SRS2797100) were selected, respectively. The t-SNE cluster visualization results were obtained by clicking on “view” and “t-SNE (interactive)”. The visualization of expression localization of genes in the tissue cells were got by entering identified hub genes in “Overlay expression of the following gene” and clicking “Add Overlay”. The specific cell types and distribution patterns of these genes in the testis and ovary were obtained.

## 5. Conclusions

In this study, the potential mechanisms underlying DON-induced reproductive toxicity were systematically explored using an integrated strategy combining network toxicology, molecular docking, MD simulation, and single cell RNA-seq. Network toxicology identified 229 targets associated with DON-induced testicular injury and 319 targets associated with DON-induced ovarian injury. The hub genes related to both types of reproductive injury were consistently identified as *AKT1*, *EGFR*, *PIK3CA*, *PIK3R1*, and *SRC*. Enrichment analysis indicated that the prolactin signaling pathway, Ras signaling pathway, HIF-1 signaling pathway, and AGE–RAGE signaling pathway in diabetic complications were closely associated with testicular injury, whereas the PI3K-Akt signaling pathway, HIF-1 signaling pathway, prolactin signaling pathway, insulin signaling pathway, and AGE–RAGE signaling pathway in diabetic complications were strongly linked to ovarian injury. Molecular docking and MD simulation further supported the favorable binding of DON to the hub targets, particularly *PIK3CA*. In addition, single cell RNA scream-seq revealed cell-type-specific distributions of the hub genes in testicular and ovarian tissues and showed that these five genes were highly expressed in both the testis (SRA667709:SRS3065430) and the ovary (SRA638923:SRS2797100). Overall, these findings deepen current understanding of DON-induced reproductive toxicity and provide new insight into how foodborne toxins may affect reproductive health. This study also offers a theoretical basis for future research on the assessment and mitigation of DON-related health risks. However, as the present findings were mainly derived from computational analyses, further studies integrating DON exposure with in vitro and in vivo validation of the core targets and major signaling pathways are still required.

## Figures and Tables

**Table 1:** Degree, MCC and MNC values of the top 10 targets.

Disease	Targets	Degree	Targets	MCC	Targets	MNC
	EGFR	56	SRC	15,823,653	EGFR	56
	HSP90AA1	49	AKT1	14,740,634	HSP90AA1	48
	AKT1	48	EGFR	12,330,220	AKT1	48
DON-induced	SRC	46	PIK3CA	11,106,558	SRC	45
testicular injury	STAT3	45	PIK3R1	11,106,428	STAT3	44
	HSP90AB1	36	JAK2	10,241,936	HSP90AB1	36
	PIK3R1	36	PIK3CB	9,611,406	PIK3R1	36
	ESR1	34	PIK3CD	9,611,400	ESR1	33
	PIK3CA	32	PTPN11	9,003,276	PIK3CA	32
	NFKB1	30	HRAS	8,055,852	NFKB1	30
	EGFR	65	SRC	17,414,269	EGFR	65
	HSP90AA1	55	AKT1	14,910,282	HSP90AA1	55
	AKT1	54	EGFR	13,348,134	AKT1	54
DON-induced	STAT3	53	PIK3CA	13,113,486	STAT3	52
ovarian injury	SRC	53	PIK3R1	13,113,330	SRC	52
	PIK3R1	43	JAK2	11,906,102	PIK3R1	43
	PIK3CA	40	PIK3CB	11,160,006	PIK3CA	40
	HSP90AB1	40	PIK3CD	11,160,000	HSP90AB1	40
	ESR1	39	PTPN11	10,824,396	ESR1	37
	STAT1	37	PIK3CG	8,996,400	TLR4	36

**Table 2:** Energies and the main binding sites of molecular docking.

Gene	PDB ID	Energy (kcal/mol)	Hydrophobic Interaction	Hydrogen Bond
AKT1	1H10	−6.1	LYS39, GLU40, GLN47	LYS39, GLU40, ALA50, LEU52
EGFR	1XKK	−7.5	VAL726	LYS745, ASP855
PIK3CA	7JIU	−7.6	ILE633	ARG818, CYS838
PIK3R1	1PKS	−5.9	TRP55	TYR14, ARG18
SRC	1A07	−5.2		LYS206, ARG208

## Data Availability

The additional data supporting the manuscript are available from the corresponding author upon request.

## Abbreviations

The following abbreviations are used in this manuscript:

DON	Deoxynivalenol
PPI	Protein–protein interaction
MCC	Maximum Clique Centrality
MNC	Maximum Neighborhood Component
GO	Gene Ontology
BP	Biological processes
CC	Cellular components
MF	Molecular functions
KEGG	Kyoto Encyclopedia of Genes and Genomes
MD	Molecular dynamics simulation
MM/GBSA	Molecular mechanics/generalized born surface area
RMSD	Root mean square deviation
RMSF	Root mean square fluctuation
Rg	Radius of gyration
SASA	Solvent accessible surface area
